# Association between conflict intensity and health outcomes in contemporary conflicts, while accounting for the vulnerability and functioning of healthcare services

**DOI:** 10.1186/s13031-025-00654-4

**Published:** 2025-03-10

**Authors:** Siddarth Daniels David, Anneli Eriksson

**Affiliations:** Department of Global Public Health, Karolinska Institutet, Stockholm, Sweden

**Keywords:** Conflict, Health, Communicable diseases, Non-communicable diseases, Vulnerability, Functioning of healthcare services

## Abstract

**Background:**

Armed conflict can be described as human development in reverse. In addition to the direct consequences of violence, there are numerous ways in which armed conflict may have indirect effects on people’s health and well-being. Studies give varying results, and health impacts seem to differ from context to context. We aimed to determine how conflict intensity is associated with health outcomes, accounting for existing vulnerabilities and the functioning of healthcare services in countries experiencing armed conflict.

**Method:**

This study is based on panel data on conflict intensity, vulnerability, healthcare service functioning, and health outcomes in 42 conflict-affected countries between 2000 and 2019 and uses fixed-effects panel regression analysis to determine the associations between conflict intensity and health outcomes.

**Results:**

Conflict intensity was positively associated with the health outcomes included in this study. As the conflict intensity increased, the mortality and prevalence of these outcomes also increased, although this increase was not statistically significant for half the outcomes (8/16). After adjusting for the vulnerabilities and functioning of healthcare services, this positive association became significant for all health outcomes. Vulnerability and functioning of healthcare services were strong predictors of outcomes. Subgroup analysis revealed that conflict intensity was more significantly associated with outcomes in countries with high and medium vulnerability scores.

**Conclusion:**

Existing vulnerabilities and healthcare system conditions are known to impact health outcomes. The association between conflict intensity and health outcomes strengthens when existing vulnerabilities and the state of healthcare services are considered. This underscores the importance of incorporating strategies to address socioeconomic inequities and strengthen healthcare system capacity in interventions for conflict-affected regions. This also raises additional concerns for long-term negative health effects related to the increasing trend of attacks on health care in contemporary conflicts.

**Supplementary Information:**

The online version contains supplementary material available at 10.1186/s13031-025-00654-4.

## Background

Armed conflict has been described as human development in reverse [1]. Violence and attacks can have detrimental effects on people’s health and lives, resulting in death, injury, and disability [2]. In addition, a conflict increases exposure to health risks, for instance, caused by displacement, leading to poorer and more crowded living conditions and potential exposure to new infections. The vulnerability of individuals and of a society increases with the disruption of protection factors, such as access to safe water supplies, electricity, and financial stability [2, 3, 4]. Those vulnerable pre-conflict are likely even more vulnerable during conflict [2, 3, 5, 6]. Armed conflict may also impact health systems, hampering access to and the availability of health services through the destruction of services, the migration of health staff and sometimes security constraints that hinder access [7]. The disruption of health care services can be an important cause of morbidity and mortality [2, 6]. For example, the health care provision in Tigray, Ethiopia, decreased to a minimum during the recent armed conflict, leaving large parts of the population without access to health care [8]. The utilization of health services has also been shown to decrease during conflict events, as security constraints can make services unreachable [9, 10].

The disruption of routine vaccination services is a risk factor for outbreaks, as many outbreaks are preventable [2, 11]. In addition, armed conflicts are associated with outbreaks of emerging and reemerging diseases [12], and outbreaks have been more difficult to stop because of insecurity and disrupted health services [13, 14, 15, 16]. Studies suggest that armed conflict is positively associated with maternal, child, and even all-cause mortality and that the intensity of conflict is an important factor, as is distance to the active conflict [2, 17, 18].

Other studies, however, have shown inconclusive results, possibly linked to the provision of humanitarian health assistance, for instance, where poor health services predate the conflict and where violence can improve antenatal care services, possibly through international health interventions [10]. In some contexts, an association is observed between armed conflicts and increased mortality from noncommunicable diseases such as chronic ischemic heart disease or unspecified heart disease [5, 19, 20]. Risk factors for chronic diseases such as alcohol and tobacco use also tend to increase in armed conflicts [5], and negative effects on mental health have been documented in many studies [21, 22]. Hence, while numerous studies have examined the impact of armed conflicts on health, the results vary, and the extent of health impacts differs from context to context.

In this paper, we aim to investigate whether health outcomes vary with the intensity of the conflict itself while accounting for existing socioeconomic vulnerabilities and the functioning of healthcare services in countries experiencing armed conflict. Going beyond battle-related deaths, we explore how and whether different health outcomes are affected by the intensity of armed conflict together with existing vulnerabilities and healthcare services.

## Methods

### Theoretical framework

Hazard, vulnerability, and exposure are widely used as ways to determine the risk of adverse effects in any type of disaster or crisis situation [23, 24, 25, 26]. The risk of a poor health outcome related to armed conflicts can, with this logic, be explained by the conflict itself (the hazardous event), people’s exposure to the conflict and the vulnerability of a society or country.

$$ \begin{array}{l}{\rm{Assumption}}:\,{\rm{Risk}}\,{\rm{of}}\,{\rm{poor}}\,{\rm{health}}\,{\rm{outcome}}\,{\rm{ = }}\,\\{\rm{Hazard*}}\,{\rm{Exposure*}}\,{\rm{Vulnerability}}\end{array} $$

In this study, we explore whether there are patterns or changes in health outcomes at the country level that can be explained by the level of *exposure* to the conflict—the hazard—as well as *vulnerability factors*. Vulnerability refers to the characteristics and circumstances of a community, system or asset that make it susceptible to the damaging effects of a hazard. These typically include socioeconomic elements, level of development and demographic factors. In addition, we specifically assess outcomes against the functioning of health care services as an additional vulnerability factor.

Hazard was defined as armed conflicts. We included and used data for the three types of armed conflicts as defined by the Uppsala Conflict Data Program (UCDP): state-based, nonstate-based and one-sided [27]. However, state-based violence causes the vast majority of battle-related deaths. We chose to use conflict intensity as a measurement of exposure. Conflict intensity was measured as the proportion of battle-related deaths in a conflict-affected country per year.

In previous research, the authors developed a severity and needs score on the basis of recognized proxy indicators for vulnerability and exposure (the 7eed model) [23, 24]. In this study, we used the vulnerability scoring presented in the model, which was calculated on the basis of values presented by the World Bank [28]. The selection of indicators was made based on indicator availability and their ability: to characterize preexisting or underlying vulnerabilities using recognised and readily available indicators, see variables. The scoring is based on indicator values among countries defined as least developed by UNDP. The scoring ranges from 2 to 6, where higher numbers indicate greater vulnerability.

### Study design

This study analysed longitudinal panel data via regression vulnerabilities to study the effects of the conflict intensity, vulnerabilities, and functioning of healthcare services on health outcomes in conflict-affected countries between 2000 and 2019.

### Setting

This study included data from countries affected by conflicts between 2000 and 2019. The data on conflicts were from the Uppsala Conflict Data Program (UCDP) [27]. To capture countries with different conflict intensities, we identified a subset of countries that fell into at least two of the intensity categories as per the UCDP—no or low conflict (< 25 conflict-related deaths per country per year), minor conflict (25–999 deaths per country per year), and war (≥ 1000 deaths per country per year)—during each year of the 20-year study period.

The 42 conflict-affected countries included Afghanistan, Algeria, Angola, Azerbaijan, Bangladesh, Burkina Faso, Burundi, Cameroon, the Central African Republic, Colombia, the Democratic Republic of the Congo (DRC), Côte d’Ivoire, Egypt, Ethiopia, India, Indonesia, Iran, Iraq, Kenya, Kyrgyzstan, Lebanon, Libya, Mali, Mozambique, Myanmar, Nepal, Niger, Nigeria, Pakistan, Philippines, Russia, Rwanda, Somalia, South Sudan (2011 onwards), Sri Lanka, Sudan, Syria, Tajikistan, Thailand, Türkiye, Ukraine, and Yemen.

### Data

We combined different datasets on reporting conflict-related mortality, different health outcomes, vulnerabilities, and functioning of the healthcare system for our analysis. Armed conflict-related deaths were calculated via UCDP data [27]. The UCDP has complied with country-wide and year wise armed-conflict data since 1946 and has been used extensively in research. It codes deaths as low, high, and the best estimate of the number of battle-related deaths for each event based on the reliability of the reports available [29]. We extracted data of the “best estimate” of deaths from 2000 to 2019 for the study countries. The vulnerability score data were extracted from the World Bank open data. The measles vaccination data were also taken from the World Bank dataset [30]. The data for most of the study outcomes came from the Global Burden of Disease (GBD) dataset [31]. The GBD study uses data from different local, national, and international sources to produce internally consistent and statistically modelled annual estimates of mortality incidence and the incidence of different diseases by year, country, and, in some cases, by subnational region.

### Variables

The main variable for this study was conflict intensity. For this study, we defined it as the conflict-related death rate per 100,000 people. We used the rate rather than absolute conflict deaths to avoid bias stemming from the size of the conflicts with respect to the population size of the countries. We assume that a conflict localized to one or two regions in a country with a high population is unlikely to exert country-wide effects.

To capture vulnerability, we used four recognized vulnerability indicators: gross national income (GNI) per capita, under-5 mortality, stunting and the adult literacy rate. The score comprises gross national income (GNI) per capita (using the atlas method based on current USD), the literacy rate (percentage of people aged 15 years and above who are literate), and under 5-year mortality (per 1000 live births), which is defined as the probability that a child born in a specific year or period will die before reaching the age of 5 years and the prevalence of stunting (height for the age of children under 5 years). These indicators are recognised as proxy indicators for various aspects of vulnerability. While GNI per capita largely gives an indication of a country’s economic vulnerability, the three under five mortality, stunting and adult literacy give indication of the level of poverty, access to health care, access to food and access to and level education in a population. Each country was scored for each year of the study period (Supplementary Materials 1). We used the measles vaccination coverage rate (percent of children aged 12 and 23 months) as a proxy indicator for the functioning of healthcare services, as suggested, for example, by Bos and Batson [32].

### Outcomes

To make the study representative of the spectrum of health outcomes, we used prevalence and mortality rates for different reproductive and child health (RCH), infectious, and noncommunicable diseases for each year during the study period. The RCH outcomes included neonatal mortality, under 1-year crude mortality, crude mortality under 5-year crude mortality, and maternal mortality. For estimates of malnutrition, we included mortality and the prevalence of acute malnutrition, presented as PEM (protein–energy malnutrition) according to the Global Burden of Disease. Infectious disease outcomes included mortality and the prevalence of diarrheal diseases, mortality and the prevalence of tuberculosis (TB), and mortality and the prevalence of HIV/AIDS. Noncommunicable diseases included mortality and cardiovascular diseases and mortality and the prevalence of diabetes mellitus.

### Statistical methods

We described the study sample by presenting the graphical time trends in the conflict-related mortality rate across the study countries. We performed fixed-effects panel regression analysis to study the relationships between different health outcomes and the conflict-related death rate, vulnerability, and functioning of healthcare services. It was estimated via the following equation:

$$\begin{array}{l}\:{\rm{ln}}\left( {Health\:outcome} \right) = \:\beta {\:_{0\:}} + \beta {\:_1}Conflict\:intensit{y_{it}}\: + \:\:\\\:\beta {\:_2}Vulnerabilit{y_{it}}\: + \:\beta {\:_3}\:Functioning\:of\:\\healthcare\:services{\:_{it}}\: + \:i + t + {u_{it}}\:\end{array}$$

Here, i denotes the country, t denotes the year, and u is the error term denoting unexplained variation in the model. We chose fixed-effects model over random-effects model given the likelihood that the error term was correlated with the explanatory variables in this study. This was confirmed by the Hausman test [33]. The use of a fixed-effects model controls for country-level factors that are time-invariant and computes associations within countries. As the outcomes were proportions, mortality rates and prevalence rates, the outcomes were log-transformed for the regression analysis. Consequently, the estimated coefficient would denote the percent change in the log outcome associated with a one-unit increase in the variable. Therefore, the percentage increase in the outcome can be expressed by the exponent of the coefficient and is calculated as $\:100\:\times\:\left({e}^{\beta\:}-1\right)$. We estimated 95% confidence intervals and denoted associations with a *p* value of less than 0.05 as statistically significant.

The sources used had more or less complete data for all the outcomes and variables for the 42 countries for the study period. The only unavailable data were for the conflict-related deaths and measles immunization rates for South Sudan between 2000 and 2010, as the country was only formed in 2011. This corresponded to approximately 2.5% missing data in the dataset. As it represented a small proportion of the whole dataset, we did not handle it specifically. We also assessed collinearity via the variance inflation factor (VIF). The VIF measures how much the variance of an estimated regression coefficient is increased due to collinearity—when there is a correlation between two or more of the independent variables. We found no collinearity between the three explanatory variables. For the vulnerability score, missing data was handled either by using the same score from previous estimation until a new estimate was reported (stunting and adult literacy). For GNI per capita, alternive sources were consulted. We also performed subgroup analysis on the basis of the vulnerability score per country per year: low (1–2), moderate (3–4), and high (5–6). Additionally, we performed a sensitivity analysis to compare the performance of our conflict-intensity metric with another conflict-intensity measure—the conflict intensity score of the Bertelsmann Transformation Index (BTI)—that reports nation-level conflict intensity. The statistical software R was used for all statistical analyses [34].

### Ethics

The data used for analysis in this study came from publicly available datasets without any personal or identifying information. Therefore, no ethical permissions were required for conducting this study.

## Results

During the study period 2000 to 2019, there were 660 conflict events in the 42 study countries. Out of these one-fifth (118) were low conflict events, half (349) were minor conflicts, and one-fourth (193) were wars. A brief description of the frequency of the data is given in Table 1. Countries in conflict had on average around 325 conflict-related deaths per 100,000 per year during the study period.

**Table 1:** Description of data used in the study

Characteristics	Frequency
Conflict-related deaths per 100,000 (median, IQR)	324.7 (10.6, 935.6)
Conflict exposure^1^ (frequency)	
No-conflict	169
Low (1 to 25 deaths per year)	118
Minor (25 to 999 deaths per year)	349
War (1000 and above deaths per)	193
Vulnerability Score (median, IQR)	4.05 (2.5,5.5)
Measles vaccination coverage in percent (median, IQR)	81 (65,94)

^1^Based on the number of conflict-related deaths per country-year: low > 25; ≥ 25; minor conflict, 25–999; war, ≥ 1000. IQR: interquartile range

**Fig. 1:**
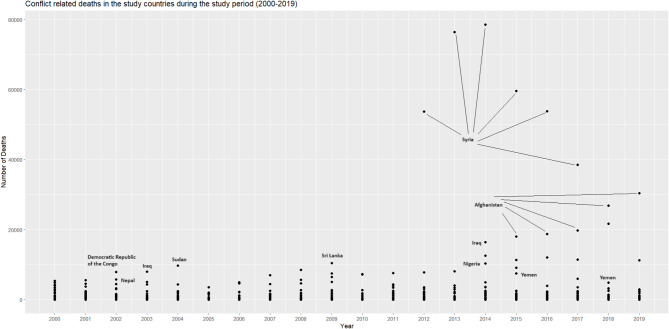
Conflict related deaths during the study period in the study countries

**Fig. 2:**
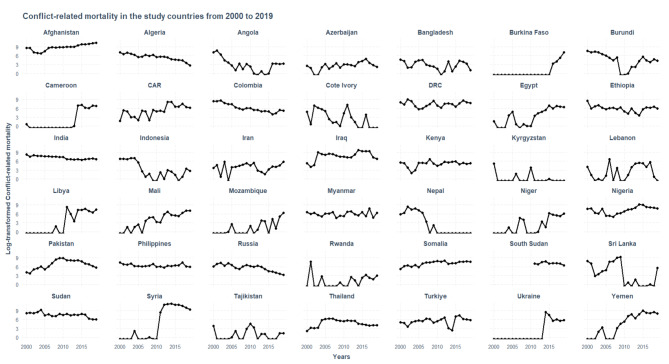
Trends in conflict-related deaths (log-transformed) in the study countries during the study period

The year 2014 recorded the highest number of deaths, followed by 2013 and 2015 (Fig. 1). Syria, Afghanistan, and Yemen contributed to high deaths in that period (Fig. 2).

### Health outcomes

**Fig. 3:**
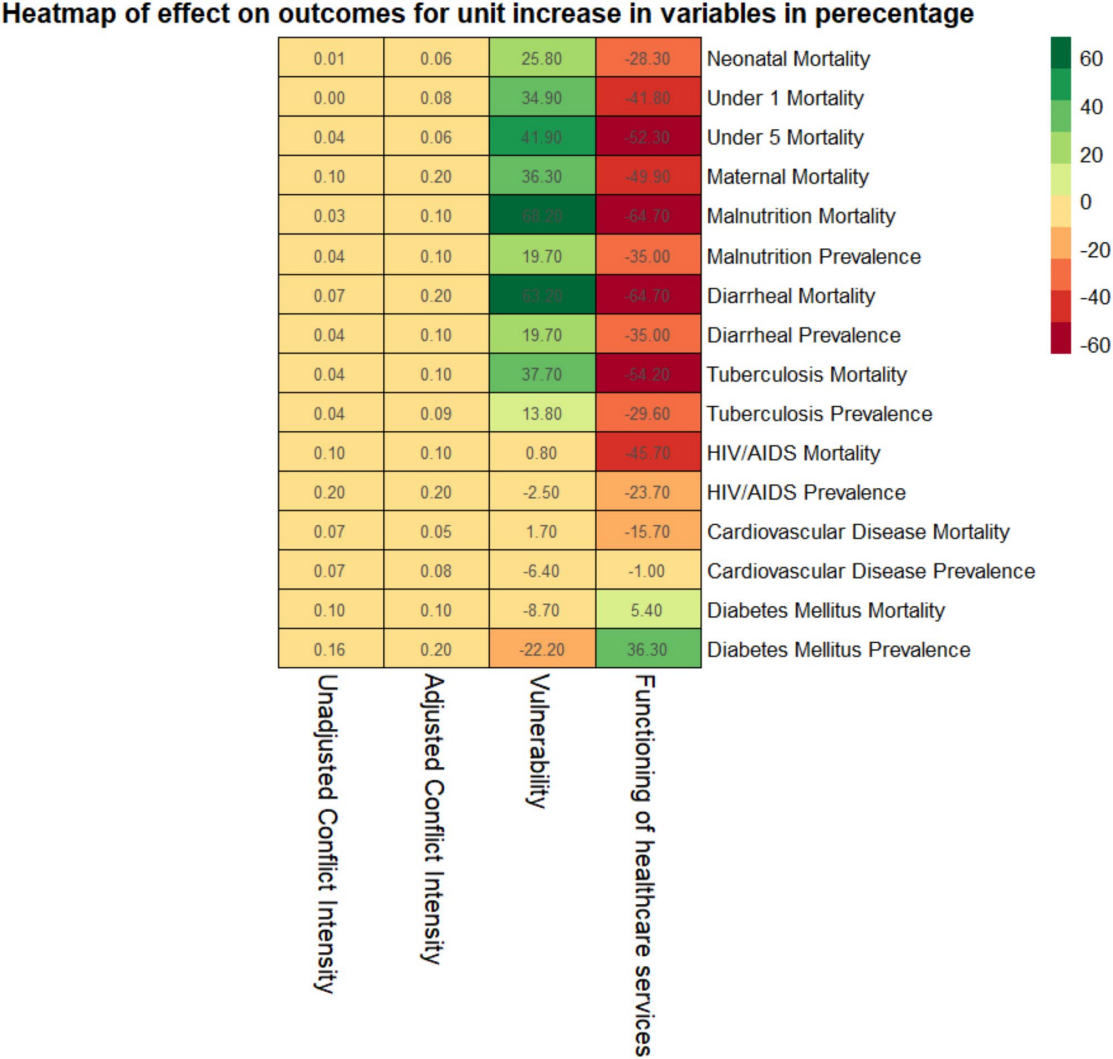
Heatmap showing the percentage change in outcomes for a one-unit change in the variables

The results of the multivariate regression for the 42 included countries (Table 2), indicate conflict intensity was positively associated with all the health outcomes. This means that as the conflict intensity increased the mortality and prevalence of these outcomes also increased. The increase was small: 0.01 to 0.2 per cent increase in the rates of the outcomes for one-unit increase in conflict intensity. (Fig. 3). Moreover, these associations were not statistically significant for half of the outcomes. After adjusting for socioeconomic vulnerabilities and functioning of healthcare services, the positive association between the outcomes and conflict intensity became stronger, that is the mortality and prevalence increased. Again, the increase was small: 0.06 to 0.2 per cent. But the association was significant for all the 16 outcomes.

**Table 2:** Association between health outcomes and conflict intensity before and after adjusting for socioeconomic vulnerabilities and the functioning of healthcare services

Outcomes	Exponential transformed estimates (95%CI)			
	Unadjusted model	Adjusted model		
	Conflict intensity	Conflict intensity	Vulnerability	Functioning of healthcare services
**Reproductive and child health outcomes**				
Neonatal mortality	1.00001 (0.9994, 1.0006)	1.0006* (1.0001, 0.001)	1.258*** (1.221, 1.284)	0.711** (0.618, 0.810)
mortality	1.00003 (0.993, 1.0007)	1.0008* (1.0003, 1.001)	1.349** (1.309, 1.377)	0.582** (0.501, 0.67)
Under5 mortality	1.0004 (0.9997, 1.001)	1.0006* (1.0001, 1.001)	1.419*** (1.377, 1.462)	0.477*** (0.406, 0.565)
Maternal mortality	1.0001* (1.0003, 1.002)	1.002** (1.001, 1.003)	1.363*** (1.323, 1.404)	0.501*** (0.423, 0.594)
**Nutrition-related outcomes**				
Malnutrition mortality	1.0003 (0.999, 1.001)	1.001** (1.0007, 1.002)	1.682*** (1.599, 1.768)	0.353*** (0.267, 0.463)
Malnutrition prevalence	1.0004 (0.999, 1.0008)	1.001** (1.0006, 1.001)	1.197** (1.161, 1.20)	0.65** (0.588, 0.733)
**Communicable disease outcomes**				
Diarrheal disease mortality	1.0007 (0.9997, 1.002)	1.002** (1.002, 1.003)	1.632*** (1.552, 1.682)	0.402*** (0.323, 0.506)
Diarrheal disease prevalence	1.0008** (1.0003, 1.001)	0.001** (1.0009, 1.002)	1.173** (1.15, 1.197)	0.740** (0.663, 0.818)
TB mortality	1.0004 (0.99, 1.0005)	1.001** (1.0009, 1.002)	1.377*** (1.336. 1.419)	0.458*** (-0.386, 0.543)
TB prevalence	1.0004* (1.0001, 1.0008)	1.0009** (1.0006, 1.001)	1.138*** (1.116, 1.15)	0.704** (0.625, 0.794)
HIV/AIDS mortality	1.001 (0.9999, 1.003)	1.001 (0.9995, 1.002)	1.008 (0.917, 1.072)	0.543* (0.165, 0.86)
HIV/AIDS prevalence	1.002* (1.0003, 1.003)	1.002* (1.009, 1.003	0.975* (0.968, 0.982)	0.763 (0.527, 1.10)
**Noncommunicable disease outcomes**				
Cardiovascular diseases mortality	1.0007* (1.0004, 1.001)	1.0005** (1.0002, 1.0008)	1.017* (1.002, 1.377)	0.843** (0.771, 0.913)
Cardiovascular diseases prevalence	1.0007** (1.0004, 1.0009)	1.0008** (1.0005, 1.001)	0.936** (0.923, 0.946)	0.99 (0.932, 1.052)
Diabetes mellitus mortality	1.001* (1.0007, 0.002)	1.001** (1.0008, 1.001)	0.915** (0.895, 0.941)	2.054 (0.935, 1.233)
Diabetes mellitus prevalence	1.0016** (1.001, 1.002)	1.002** (1.001, 1.002)	0.778** (0.763, 0.802)	1.363** (1.197, 1.552)

* *p* value > 0.05; ‘**’ *p* value > 0.01 **

We also observed that socioeconomic vulnerabilities and functioning of healthcare service had much stronger association with the outcomes, resulting in changes of up to 67 per cent increase in the rates of the outcomes per one-unit increase in these co-variates. (Fig. 3) in the outcomes. The outcomes for reproductive and child health, malnutrition, and communicable disease were positively associated with the vulnerability These associations were statistically significant as well. Indicating, that as vulnerability increased the mortality and prevalence of these diseases increased. However, this positively association reduced and, in some cases, reversed for noncommunicable diseases like cardiovascular diseases and.

diabetes where mortality and prevalence of these diseases showed either a slight increase or a decrease as the vulnerability increased. In contrast, functioning of healthcare services was negatively and significantly associated with reproductive and child health, malnutrition, and communicable disease outcomes. This negative association reduced or was positive for non-communicable diseases.

**Fig. 4:**
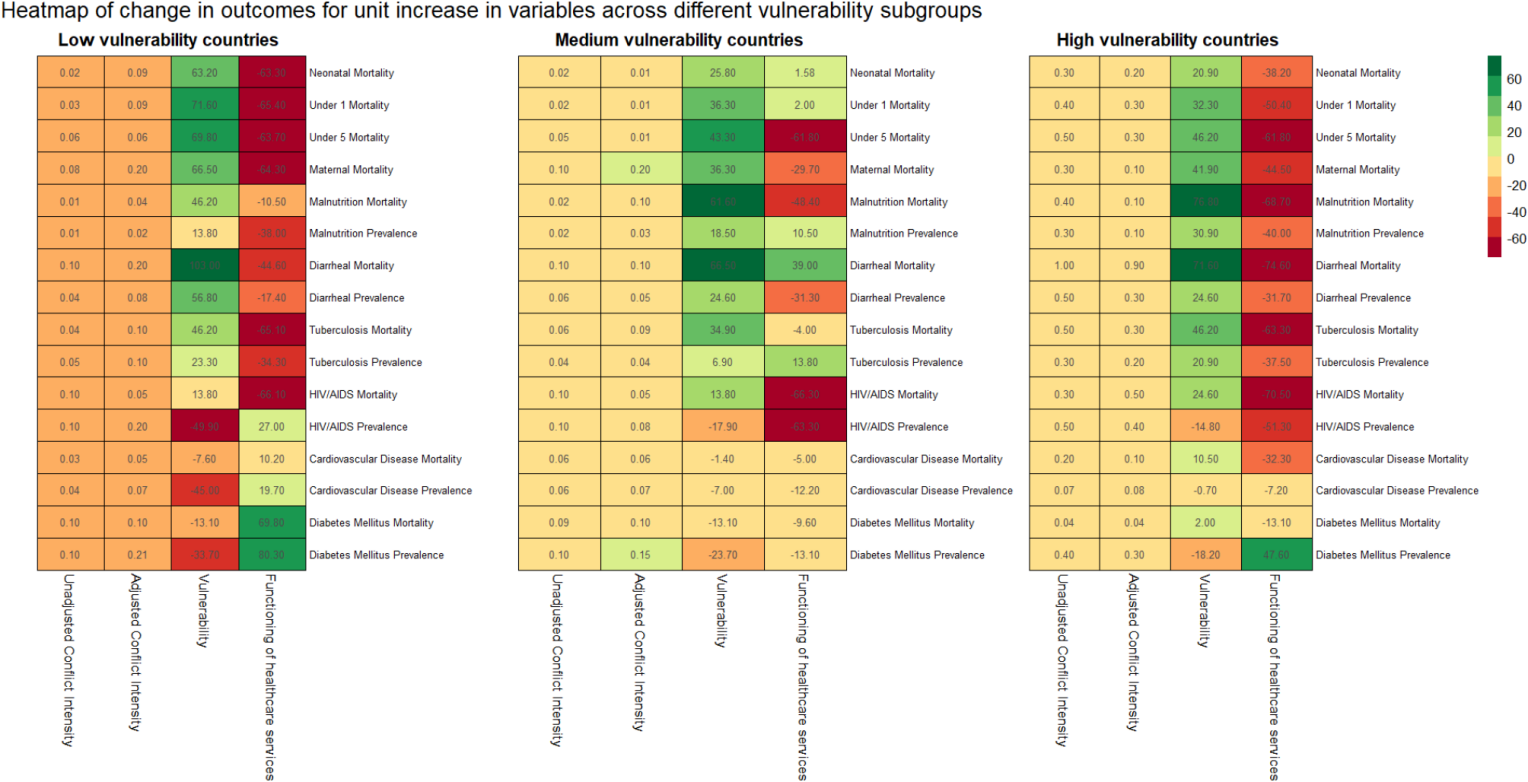
Heatmap showing the percentage change in outcomes for a one-unit change in the variables across different vulnerability subgroups

After subgrouping the countries by vulnerability score into low, medium, and high categories for each year of the study period, the analysis revealed similar trends (Table 3). The study outcomes revealed positive associations with conflict intensity across the vulnerability categories. The effect of conflict intensity on the outcomes increased, and its significance increased as the vulnerability score increased. Conflict intensity was a significant factor in countries with high and medium vulnerability scores. After adjusting for socioeconomic vulnerabilities and the functioning of healthcare services, this positive association became stronger in countries with high and medium vulnerability scores. The increase was small: 0.01 to 0.2% increase in the rates of the outcomes per-unit increase in conflict intensity. (Fig. 4). These associations were not observed for countries with low vulnerability scores.

**Table 3:** Association between health outcomes and conflict intensity across different subgroups of vulnerability before and after adjusting for socioeconomic vulnerabilities and healthcare service functioning

Outcomes	Vulnerability level		Exponential transformed estimates (95%CI)			
		Unadjusted model	Adjusted Model			
		Conflict-intensity	Conflict-intensity	Vulnerability	Functioning of healthcare services	
**Reproductive and child health outcomes**						
Neonatal mortality	Low	1.0002 (0.998, 1.002)	1.0009 (0.997, 1.0007)	1.632** (1.336, 2.103)	0.367** (0.236, 0.565)	
	Medium	1.0002 (0.9998, 1.0007)	1.00009 (0.999, 1.0004)	1.258** (1.209, 1.296)	0.1358 (0.886, 1.390)	
	High	1.003*** (1.002, 1.005)	1.002** (1.001, 1.003)	1.209*** (1.161, 1.246)	0.618*** (0.565, 0.670)	
Under1 mortality	Low	1.0003 (0.999, 1.002)	1.0009 (0.998, 1.001)	1.716** (1.377, 2.138)	0.346** (0.216, 0.548)	
	Medium	1.0002 (0.9996, 1.0008)	1.00007 (0.9995, 1.0004)	1.363** (1.309, 1.433)	1.02 (0.755, 1.363)	
	High	1.004** (1.002, 1.006)	1.003*** (1.001, 1.004)	1.323** (1.258, 1.377)	0.496*** (0.436, 0.565)	
Under5 mortality	Low	1.0006 (0.999, 1.003)	1.0006 (0.999, 1.001)	1.698** (1.336, 2.159)	0.364** (0.199, 0.554)	
	Medium	1.0005 (0.999, 1.001)	1.0001 (0.999, 1.0007)	1.433** (1.363, 1.506)	0.382** (0.618, 1.221)	
	High	1.005** (1.002, 1.007)	1.003** (1.001, 1.004)	1.462** (1.377, 1.552)	0.382** (0.323, 0.453)	
Maternal mortality	Low	1.0008 (0.987, 1.003)	1.002 (-0.9997, 1.004)	1.665** (1.309, 2.117)	0.357** (0.220, 0.600)	
	Medium	1.001** (1.0008, 1.002)	1.002** (1.001, 1.003)	1.363** (1.284, 1.447)	0.704 (0.472, 1.040)	
	High	1.003* (1.0008, 1.005))	1.001 (0.9993, 1.003)	1.419** (1.323. 1.506)	0.554** (0.463, 0.670)	
**Nutrition-related outcomes**						
Malnutrition mortality	Low	1.0001 (0.999, 1.001)	1.0004 (-0.999, 1.001)	1.462* (1.221. 1.75)	0.895 (0.631, 1.296)	
	Medium	1.0002 (0.999, 1.001)	1.001 (0.9998, 1.005)	1.616** (1.462, 1.786)	0.516 (0.269, 1.116)	
	High	1.004 (0.991, 1.0009)	1.001 (0.995, 1.003)	1.768*** (1.521, 2.054)	0.313** (0.1353, 0.307)	
Malnutrition prevalence	Low	1.0001 (0.9992, 1.0005)	1.0002 (0.9991, 1.0004)	1.138** (1.051, 1.246)	0.962 (0.810, 1.138)	
	Medium	1.0002 (0.9993, 1.0001)	0.0003 (0.9993, 1.0003)	1.185** (1.138, 1.221)	1.105 (0.869, 1.03)	
	High	1.003* (1.001, 1.004)	1.001* (1.0003, 1.002)	1.309** (1.258, 1.363)	0.600** (0.537, 0.670)	
**Communicable disease outcomes**						
Diarrheal disease mortality	Low	1.001 (0.997, 0.0005)	1.002* (1.0004, 1.004)	2.033** (1.616, 2.534)	0.554* (0.346, 0.895)	
	Medium	1.001* (1.0005, 1.002)	1.001** (1.0004, 1.02)	1.665** (1.521, 1.803)	1.390 (0.755, 2.559)	
	High	1.01** (1.007, 1.02)	1.009** (1.006, 1.116)	1.716** (1.584, 1.877)	0.254** (0.199, 0.326)	
Diarrheal disease prevalence	Low	1.0004 (0.999, 1.0006)	1.0008 (0.9982, 1.0009	1.568** (1.404, 1.750)	0.826 (0.637, 1.476)	
	Medium	1.0006* (1.0001, 1.0008)	1.0005* (1.00006, 1.001	1.246*** (1.185, 1.296)	0.687** (0.945, 0.501)	
	High	1.005** (1.003, 1.006)	1.003** (1.002, 1.004)	1.246** (1.197, 1.296)	0.683** (0.606, 0.763)	
TB mortality	Low	1.0004 (0.998, 1.001)	1.001 (0.9996, 1.003)	1.462*** (1.173, 1.84)	0.349** (0.214, 0.565)	
	Medium	1.0006 (0.9999, 1.001)	1.0009** (1.0002, 1.01)	1.349*** (1.284, 1.419)	0.96 (0.657, 1.419)	
	High	1.005** (1.002, 1.008)	1.003** (1.001, 1.005)	1.462** (1.377, 1.537)	0.367** (0.31, 0.431)	
TB prevalence	Low	1.0005 (0.999, 1.0003)	1.001** (1.0001, 1.002)	1.233*** (1.116, 1.349)	0.657** (0.532, 0.802)	
	Medium	1.0004** (1.0001, 1.0006)	1.0004** (1.0001, 1.0006)	1.069** (1.04, 1.083)	0.138 (0.990, 0.323)	
	High	1.003** (1.002 1.004)	1.002** (1.001, 1.003)	1.209** (1.173, 1.246)	0.625** (0.75, 0.462)	
HIV/AIDS mortality	Low	1.001 (0.9996, 1.003)	1.0005 (0.9992, 1.001)	1.138* (1.02, 1.258)	0.339* (0.148, 0.794)	
	Medium	1.001** (1.0001, 0.002)	1.0005 (0.9992, 1.001)	1.138* (1.02, 1.284)	0.337** (0.149, 0.771)	
	High	1.003 (0.997, 1.01)	1.005 (-0.998, 1.012)	1.246 (0.98, 1.584)	0.295** (0.146, 0.594)	
HIV/AIDS prevalence	Low	1.001 (0.999, 1.004)	1.002** (1.00001, 1.005)	0.501** (0.364, 0.69)	3.706** (1.877, 7.315)	
	Medium	1.001** (1.0004, 0.002)	1.0008 (0.999, 1.001)	0.802** (0.733, 0.878)	0.367** (0.182, 0.657)	
	High	1.005* (1.002, 1.011)	1.004 (0.999, 1.01)	0.852 (0.697, 1.040)	0.481* (0.269, 0.86)	
**Noncommunicable disease outcomes**						
Cardiovascular diseases mortality	Low	1.0003 (0.999, 1.001)	1.0005 (0.9999, 1.001)	0.924* (0.86, 0.997)	1.102 (0.94, 1.284)	
	Medium	1.0006* (1.0002, 1.0009)	1.0006* (1.0003, 1.0009)	0.985 (0.956, 1.01)	0.995 (0.81, 1.221)	
	High	1.002* (1.001, 1.003)	1.001** (1.0008, 1.002)	1.105** (1.075, 1.138)	0.677** (0.618, 0.748)	
Cardiovascular diseases prevalence	Low	1.0004 (0.9996, 0.001)	1.0007 (0.999, 1.0014)	0.755** (0.69, 0.818)	1.197* (1.105, 1.433)	
	Medium	1.0006* (1.0004, 1.0009)	1.0007** (1.0005, 1.0009)	0.93** (0.911, 0.950)	0.878 (0.852, 1.138)	
	High	1.0007** (1.0003, 1.001)	1.0008** (1.0006, 1.001)	0.993 (0.979, 1.007)	0.928** (0.895, 0.968)	
Diabetes mellitus mortality	Low	1.001 (0.9993, 1.0029)	1.001 (0.999, 1.003)	0.869 (0.835, 1.072)	*1.698 (1.070, 2.691)	
	Medium	1.0009* (1.0005, 1.0013)	1.001** (1.0006, 1.001)	0.869** (0.835, 0.904)	0.904 (0.69, 1.17)	
	High	1.00039 (0.99996, 1.001)	1.0004 (0.9996, 1.001)	1.020 (0.990, 1.050)	0.869* (0.802, 0.942)	
Diabetes mellitus prevalence	Low	1.001 (0.9998, 1.003)	1.0021** (1.0006, 1.0037)	0.663*** (0.554, 0.794)	1.803** (1.221, 2.664)	
	Medium	1.001** (1.0007, 1.002)	1.0015** (1.001, 1.002)	0.763** (0.726, 0.794)	0.869 (0.625, 1.197)	
	High	1.004** (1.0027, 1.0058)	1.003 (1.0019, 1.004)	0.818** (0.786, 0.843)	1.476** (1.323, 1.648)	

* *p* value > 0.05; ** *p* value > 0.01 **

The vulnerability and functioning of healthcare services were strong and significant predictors of health outcomes across all subgroups. As seen previously, outcomes for reproductive and child health, nutrition, and communicable disease were positively associated with the covariates of vulnerability and negatively associated with the functioning of healthcare services. Similarly, for noncommunicable diseases, the trend of outcomes being negatively associated with increased vulnerability and positively associated with improved functioning of healthcare services continued, except for HIV/AIDS prevalence.

The sensitivity analysis using the Conflict Intensity Score of the BTI showed the regression coefficients and model parameters were similar across almost all variables (Supplementary Material 2). Only the regression coefficients for conflict intensity were different. This could be because the ranges for the two measures of conflict intensity—conflict-related death rate per 100,000 individuals and BTI’s Conflict Intensity Score—have different scales. Conflict-related death rate per 100,000 individuals in the study sample ranged from 0 to 391 while the BTI’s Conflict Intensity Score ranged from 2 to 10 in the analysis sample. Therefore, every unit change in the model using BTI’s Conflict Intensity Score meant a larger change in the outcome measure and a larger coefficient compared to our model. However, they had similar *p*-values. Given that other regression coefficients for the other variables and model-fit parameters are similar, the conflict-intensity measure used in this study performs comparable to that of the Conflict Intensity Score of the BTI.

## Discussion

This study analysed the associations between different health outcomes and the intensity of the conflict adjusted for existing vulnerability and the functioning of healthcare services. We found that all outcomes—reproductive and child health, nutrition, communicable, and noncommunicable diseases—were positively associated with conflict intensity. The association was, however, not significant for half the outcomes. After adjusting for vulnerability and the functioning of healthcare services, the associations between conflict intensity and outcomes were significant. This was especially true for countries with high and medium vulnerability. The effect of conflict intensity on the change in the rates of the outcomes was small. On the other hand, almost all outcomes were significantly associated with the vulnerability and functioning of healthcare services. In our analysis, it appears that conflict intensity by itself does not always affect health outcomes in conflict-affected countries and that the effect, if any, is quite small. The role of conflict intensity becomes more relevant in countries with low and medium vulnerability statuses. In fact, the vulnerability and functioning of healthcare services may be stronger determinants of health outcomes than conflict intensity in these settings.

Conflict intensity has been shown to affect health [13, 17, 35, 36, 37]. The absence of a strong significant relationship between conflict intensity and all health outcomes indicates that the mechanisms underlying the associations between conflict intensity and health outcomes are complex and vary across outcomes. It is also possible that measures of conflict intensity other than conflict-related deaths, such as the frequency or duration of conflict, may have a stronger association with health outcomes. A larger sample size may also yield a stronger association between conflict-related deaths and health outcomes.

There is also evidence that certain health outcomes tend to remain unchanged or even perform better in conflict-affected regions because they provide better healthcare services from humanitarian aid organizations working in relief camps and among displaced communities than local government services do. For example, skilled birth attendance, facility deliveries, and immunizations increased linearly in Afghanistan during the conflict period of 2003–2015 [38]. Healthcare services in conflict-affected areas of South Sudan are concentrated around relief camps [39]. In Syria, humanitarian organizations were able to use innovative mechanisms to meet the emerging needs of conflict-affected populations and provide accessible services to even remote populations [40]. In Northern Uganda, better maternal health service delivery was observed during the conflict period because of interventions by humanitarian organizations; however, post-conflict, health indicators decreased after the cessation of these interventions, as people were once again dependent on local healthcare services where basic resources were poor, such as before the conflict [41].

Our results indicate that conflict intensity was significantly associated with health outcomes in the high- and medium-level vulnerability status countries. Hence, in settings with lower levels of vulnerability, better socioeconomic conditions and healthcare functioning can buffer against the adverse effects of conflict intensity. Research from Colombia has shown that while there are some differences in reproductive and child health outcomes between low- and high-intensity conflict municipalities, continued stable resource allocation and trained healthcare workers help alleviate the effects of armed conflict without affecting access to care or mortality [42]. Similarly, it has been noted that maternal health outcomes in the Palestinian Territories were adequate despite the ongoing conflict (prior to the 2023 war) [43].

All the health outcomes in this study, with the exception of HIV/AIDS incidence, were positively associated with vulnerability. The role of existing vulnerabilities in leading to poor health outcomes has been well established in the literature. All four indicators that are part of our vulnerability index have been shown to negatively affect health outcomes. Regions with low income levels continue to have inadequate maternal and child health outcomes, poor nutritional outcomes, and a high prevalence of communicable diseases [44, 45, 46]. Similarly, poor levels of literacy, indicating poor health literacy, are another determinant of poor health outcomes [47, 48, 49, 50]. Higher child mortality and stunting are also proxies for overall poor health outcomes [51, 52, 53].

The negative association with HIV/AIDS incidence could be because of reduced resources and accessibility to test and report new cases or increased mortality due to the limited availability of treatment as a consequence of increasing existing vulnerabilities. Similarly, the functioning of healthcare services was negatively associated with communicable disease outcomes, i.e., improvements in the functioning of healthcare services were associated with reduced prevalence and mortality related to reproductive and child health and communicable diseases. This is expected, as robust healthcare services can address the burden of these diseases through better care and treatment.

In our analysis, vulnerability was negatively associated with noncommunicable health outcomes. The detection and prevalence of cardiovascular diseases and diabetes increase as socioeconomic conditions improve. Better living conditions, improved sanitation and nutrition, and greater access to healthcare contribute to the reduction of communicable diseases and improved knowledge and surveillance of noncommunicable diseases [54, 55, 56]. This likely explains the negative association of vulnerability with noncommunicable health outcomes. Additionally, it would also explain the positive association between the functioning of healthcare services and noncommunicable disease in our analysis. The small positive association between the functioning of healthcare services and the mortality rate due to cardiovascular diseases could be because of the general trend toward a substantial increase in deaths due to this condition in medium- and high-resource settings [57].

Our analysis of conflict-affected countries indicates that the vulnerability and functioning of healthcare services are stronger determinants than the conflict intensity of health outcomes. This study does not assess how conflict intensity affects the vulnerability and functioning of health care services. However, previous studies have shown that armed conflict severely affects healthcare services and leads to stagnation or deterioration of socioeconomic conditions and other factors associated with vulnerability [58, 59, 60]. Thus, the indirect effects of armed conflicts have a negative effect on health outcomes over time, whereas the degree of conflict intensity fluctuates from year to year. This fluctuation does not necessarily lead to similar fluctuations in health outcomes. A study from the Gaza Strip reported that continuously living in conflict-affected areas reduced incomes and access to healthcare facilities, which led to negative health outcomes even among those not exposed to violence [61]. Similarly, a study comparing conflict-affected regions in Mali, Cameroon, Nigeria, and the DRC revealed that primary healthcare services remained poor even in areas with no or low-intensity conflict, with socioeconomic disparities determining the quality of services regardless of conflict intensity [62]. Even post-conflict countries continue to experience vulnerabilities and challenges to health services beyond conflict violence, given that it takes considerable time and resources to rebuild societal structures and service delivery networks [63, 64].

Our research highlights the complex nature of armed conflict and the interplay of different factors, such as conflict intensity, existing socioeconomic vulnerabilities and the state of health services and services, that could influence health outcomes. The increasing trend of attacks on health care in contemporary conflicts is therefore, in addition to violating the protection of health establishments, according to International Humanitarian Law (IHL), a serious long-term threat to the health of people in armed conflicts [65]. While it is important to focus on the impact of immediate violence and displacement due to armed conflicts, the possibly stronger, more vicious socioeconomic vulnerability and healthcare service functions that augment the detrimental impact of conflicts must be factored in. Addressing existing socioeconomic inequities in a region and strengthening local healthcare services to deliver sustainable and equitable services post-conflict has been recognized as part of the humanitarian response in conflict-affected areas to improve health outcomes [66, 67, 68]. It has long been recognized that such initiatives can play a role in peace-building, yet there is still a significant gap in addressing factors related to vulnerability as part of conflict prevention [69, 70, 71]. We believe that our study findings support this.

This study on the associations between different reproductive and child health, nutritional, communicable disease, and noncommunicable disease outcomes and the conflict intensity adjusted for vulnerability and healthcare service functioning has several limitations. Our data sources may not be comprehensive in capturing the outcomes and variables. The UCDP uses news reports to compile conflict-related deaths and may have missed events and incidents not captured in reports. The GBD is modelled on estimates using available governmental, nongovernmental, and research data, which could be incomplete or have reporting inconsistencies, especially in conflict-affected settings, leading to a conservative estimate of mortality and the prevalence of different disease conditions. Similarly, the World Bank datasets are prone to the same limitations. Nevertheless, to our knowledge, these sources are some of the best at providing data on the variables and outcomes for the selected countries during the study period.

We used conflict-related deaths per 100,000 population as a measure for conflict-intensity in this study. We realise that this measurement doesn’t give as the full spectrum of for instance forced displacement, and other types of warfare. Conflict related deaths are however, commonly used as a way to estimate conflict intensity [72]. Using this metric, gives regression coefficients are small—corresponding to change in rate of the outcomes for one additional conflict-related death per 100,000 population. Consequently, the effect-sizes are small and may be difficult to always observe in the field and must be kept in mind while interpreting the absolute values of the regression coefficients. However, we used this measure as a standardized way to compare conflict intensity across different countries accounting for variations in population size and the extent of the population-affected by the conflict in the countries. Also, our analysis using this measure does give an overall picture of the association between conflict-intensity and health outcomes, which was smaller and weaker than healthcare services functioning and socioeconomic factors. Moreover, our sensitivity analysis of this measure with other measures of conflict intensity indicate that it does point in the same direction. It would be difficult to quantify the absolute effect of conflict-intensity on health outcomes using conflict-related deaths per 100,000 population as a measure. But this is beyond the scope of this study and such analysis would require a more appropriate measure of intensity.

We have measured the outcomes and variables at the country level, which could conceal conflicts concentrated in subnational areas that do not largely affect the country as a whole. For example, in Türkiye, the majority of conflict-related deaths were reported from the Kurdistan region. The health outcomes and variables were also measured at the national level, masking inequalities and patterns in these conflict-affected regions. However, there are few publicly available sources for yearly data on health outcomes and variables at the regional level for each conflict-affected region. The data may also have missed refugee populations displaced across borders in neighbouring countries, which have not been included in our cohort of countries. Moreover, we used a sample of 42 countries for a period of 20 years for a fixed set of health outcomes in this analysis. The findings may not be generalizable to all conflict-affected countries beyond the study period for a range of health outcomes. Detection bias of the outcomes is another limitation of the data used in this study [73]. For example, for noncommunicable diseases, reporting bias may exist due to inadequate knowledge of noncommunicable diseases affecting screening and detection [74].

We used fixed-effects panel regression in this analysis, as the Hausman test indicated. A fixed-effects model does not consider time-variant variables. There could be factors that affect health outcomes, such as the nature of humanitarian aid, duration of conflict, and political structures in these countries. However, what these are beyond the scope of this paper should be explored in future research. The use of panel regression models is susceptible to missing variables that were not included in the analysis, which could explain changes in health outcomes. We have tried to use a broad vulnerability score that includes different aspects of vulnerability to the impacts of hazards. Immunization rate has been extensively used as a proxy for the functioning of healthcare services. Nevertheless, fixed-effects panel regression provides results in relation to the country, which is more robust, rather than global averages.

Studying the associations between armed conflict, vulnerability, and the functioning of healthcare services and different health outcomes will add evidence on the health impact of armed conflict. The findings of this study can be used to push for the design of humanitarian interventions that integrate strategies to address vulnerability and strengthen local health systems. Additionally, including components to build the capacity of health sector in conflict-affected areas, beyond the immediate response, would be important to address the burden of diseases that persist post-conflict. The need for more investigations into how to achieve long-term equity and capacity-building for and sustain improvements in health in conflict areas has been identified as an area for future research [75]. On the basis of these findings, future research should examine whether other measures of intensity, such as the frequency or length of conflict, other conflict mortality datasets, and time lagged effects, yield stronger associations. It would also be important to study, with a deeper focus, the patterns of specific health outcomes and different types of conflict and other measures of vulnerability and functioning of healthcare services to better understand the impact of armed conflict on health. Additionally, further research could explore how health outcomes in conflict-affected countries vary from those in non-conflict countries and the complex indirect effects of conflict on the development of healthcare systems in these settings.

## Conclusion

Conflict intensity is positively associated with different health outcomes in countries affected by conflict. Our analysis shows that this association becomes stronger after accounting for existing vulnerabilities and the state of healthcare services. This was especially true for countries with high and medium vulnerability statuses. Moreover, these two covariates are more strongly associated with health outcomes than conflict intensity itself. The effect of conflict intensity was lower than that of existing vulnerabilities and the state of healthcare services. The findings underscore the need to integrate strategies to address socioeconomic inequities and increase the capacity of the healthcare system as part of interventions in conflict-affected areas. This also raises additional concerns about the long-term negative health effects related to the increasing trend of attacks on health care in contemporary conflicts.

## Electronic supplementary material

Below is the link to the electronic supplementary material.

Supplementary Material 1

Supplementary Material 2

## Data Availability

The datasets used and analysed during the current study are available from the corresponding author on reasonable request.
