# Supplementary material for: Association between conflict intensity and health outcomes in contemporary conflicts, while accounting for the vulnerability and functioning of healthcare services
Source: Confl Health. 2025 Mar 10;19:14. doi: 10.1186/s13031-025-00654-4 (PMC11892221; doi:10.1186/s13031-025-00654-4)
Supplement: Supplementary file 1 — Supplementary Material 1 [file 13031_2025_654_MOESM1_ESM.docx]

Sensitivity analysis of the two models with different conflict intensity measurements: Model 1 with conflict intensity as defined in this study and Model 2 with Conflict Intensity Score from Bertelsmann Transformation Index (BTI)

|  | | Model 1  (Conflict deaths per 100,000 persons) | | | Model 2  (Bertelsmann Transformation Index (BTI): Conflict intensity) | | |
| --- | --- | --- | --- | --- | --- | --- | --- |
| Outcomes | *Model parameters* | *Conflict intensity* | *Vulnerability* | *Functioning of healthcare services* | *Conflict intensity* | *Vulnerability* | *Functioning of healthcare services* |
| Reproductive and child health outcomes | | | | | | | |
| Neonatal mortality | Coefficient | 0.000422 | 0.1931 | -0.154 | 0.01526 | 0.1889 | -0.177 |
|  | Standard Error | 0.000339 | 0.0221 | 0.129 | 0.00643 | 0.0219 | 0.127 |
|  | p-value | 0.213 | 0.00** | 0.233 | 0.018* | 0.00** | 0.166 |
|  | Adjusted R-squared | 0.14235 | | | 0.15629 | | |
|  | F-statistic | 30.51 | | | 32.51 | | |
| Under-1 mortality | Coefficient | 0.000500 | 0.2508 | -0.210 | 0.01173 | 0.2469 | -0.212 |
|  | Standard Error | 0.000377 | 0.0246 | 0.144 | 0.00721 | 0.024 | 0.143 |
|  | p-value | 0.19 | 0.00** | 0.15 | 0.10 | 0.0** | 0.14 |
|  | Adjusted R-squared | 0.22 | | | 0.223 | | |
|  | F-statistic | 41.9 | | | 42.35 | | |
| Under-5 mortality | Coefficient | 0.000308 | 0.3155 | -0.222 | 0.01693 | 0.3116 | -0.260 |
|  | Standard Error | 0.000437 | 0.0285 | 0.167 | 0.00831 | 0.0283 | 0.165 |
|  | p-value | 0.481 | 0.00** | 0.183 | 0.042 | 0.00** | 0.114 |
|  | Adjusted R-squared | 0.26 | | | 0.273 | | |
|  | F-statistic | 48.98 | | | 50.92 | | |
| Maternal mortality | Coefficient | 0.00135 | 0.2683 | -0.314 | 0.03056 | 0.2579 | -0.316 |
|  | Standard Error | 0.000423 | 0.0276 | 0.161 | 0.00803 | 0.0273 | 0.159 |
|  | p-value | 0.0014* | 0.00** | 0.05175* | 0.000141* | 0.00** | 0.04664* |
|  | Adjusted R-squared | 0.21 | | | 0.22 | | |
|  | F-statistic | 40.61 | | | 42.67 | | |
| Nutrition-related outcomes | | | | | | | |
| Malnutrition mortality | Coefficient | 0.001139 | 0.4057 | -0.586 | 0.00378 | 0.4004 | -0.505 |
|  | Standard Error | 0.000711 | 0.0464 | 0.271 | 0.01369 | 0.0466 | 0.271 |
|  | p-value | 0.109 | 0.0** | 0.031* | 0.782 | 0.0** | 0.042* |
|  | Adjusted R-squared | 0.164 | | | 0.156 | | |
|  | F-statistic | 33.68 | | | 32.52 | | |
| Malnutrition  prevalence | Coefficient | 0.000833 | 0.1651 | -0.1789 | 0.01602 | 0.1591 | -0.1699 |
|  | Standard Error | 0.000249 | 0.0162 | 0.0950 | 0.00476 | 0.0162 | 0.0944 |
|  | p-value | 0.00082** | 0.00** | 0.059 | 0.00077** | 0.0** | 0.071 |
|  | Adjusted R-squared | 0.232 | | | 0.232 | | |
|  | F-statistic | 43.87 | | | 43.92 | | |
| Communicable disease outcomes | | | | | | | |
| Diarrheal disease mortality | Coefficient | 0.00213 | 0.4374 | -0.314 | 0.0318 | 0.4235 | -0.256 |
|  | Standard Error | 0.000566 | 0.0369 | 0.216 | 0.0110 | 0.0373 | 0.217 |
|  | p-value | 0.0001** | 0.00** | 0.145 | 0.003** | 0.0** | 0.237 |
|  | Adjusted R-squared | 0.30 | | | 0.288 | | |
|  | F-statistic | 56.84 | | | 53.47 | | |
| Diarrheal disease prevalence | Coefficient | 0.00118 | 0.1609 | -0.1003 | 0.01974 | 0.1529 | -0.0759 |
|  | Standard Error | 0.000298 | 0.0195 | 0.1138 | 0.00576 | 0.0196 | 0.1140 |
|  | p-value | 0.0** | 0.0** | 0.378 | 0.0** | 0.00** | 0.505 |
|  | Adjusted R-squared | 0.130 | | | 0.11 | | |
|  | F-statistic | 29.18 | | | 27.48 | | |
| TB mortality | Coefficient | 0.000715 | 0.2792 | -0.272 | 0.01544 | 0.2738 | -0.270 |
|  | Standard Error | 0.000413 | 0.0269 | 0.158 | 0.00789 | 0.0269 | 0.165 |
|  | p-value | 0.083 | 0.0** | 0.085 | 0.053 | 0.0** | 0.072 |
|  | Adjusted R-squared | 0.23 | | | 0.22 | | |
|  | F-statistic | 44.16 | | | 45.5 | | |
| TB prevalence | Coefficient | 0.000636 | 0.1277 | -0.1064 | -0.01490 | 0.1227 | -0.1094 |
|  | Standard Error | 0.000199 | 0.0130 | 0.0758 | 0.00377 | 0.0128 | 0.0746 |
|  | p-value | 0.0014** | 0.0** | 0.1607 | 0.00** | 0.0** | 0.142 |
|  | Adjusted R-squared | 0.207 | | | 0.223 | | |
|  | F-statistic | 39.86 | | | 42.43 | | |
| HIV/AIDS mortality | Coefficient | 0.000435 | 0.1651 | 0.305 | 0.0280 | 0.1713 | 0.374 |
|  | Standard Error | 0.000848 | 0.0553 | 0.324 | 0.0162 | 0.0550 | 0.320 |
|  | p-value | 0.6079 | 0.0029*8 | 0.3466 | 0.0832 | 0.0018** | 0.2430 |
|  | Adjusted R-squared | 0.136 | | | 0.123 | | |
|  | F-statistic | 3.13 | | | 4..08 | | |
| HIV/AIDS prevalence | Coefficient | 0.001183 | -0.0701 | 0.182 | 0.0459 | -0.0579 | 0.256 |
|  | Standard Error | 0.000641 | 0.0418 | 0.245 | 0.0120 | 0.0409 | 0.238 |
|  | p-value | 0.064 | 0.093 | 0.457 | 0.01* | 0.156 | 0.282 |
|  | Adjusted R-squared | 0.148 | | | 0.0988 | | |
|  | F-statistic | 2.21 | | | 6.00 | | |
| Non-communicable disease outcomes | | | | | | | |
| Cardiovascular diseases mortality | Coefficient | 0.000655 | 0.027 | -0.023205 | 0.01323 | 0.0321 | -0.0280 |
|  | Standard Error | 0.000196 | 0.0128 | 0.074861 | 0.00375 | 0.0128 | 0.0742 |
|  | p-value | 0.0009** | 0.034* | 0.756 | 0.00041** | 0.0118** | 0.70559 |
|  | Adjusted R-squared | 0.089 | | | 0.0837 | | |
|  | F-statistic | 6.77 | | | 7.218 | | |
| Cardiovascular diseases prevalence | Coefficient | 0.000706 | -0.0412 | -0.000451 | 0.01401 | -0.0361 | -0.006530 |
|  | Standard Error | 0.000156 | 0.0102 | 0.059647 | 0.00298 | 0.0102 | 0.059108 |
|  | p-value | 0.00** | 0.0** | 0.99 | 0.00** | 0.00** | 0.91 |
|  | Adjusted R-squared | 0.033 | | | 0.027 | | |
|  | F-statistic | 11.53 | | | 12.11 | | |
| Diabetes mellitus mortality | Coefficient | 0.001211 | -0.0528 | 0.0949 | 0.02620 | -0.0437 | 0.0927 |
|  | Standard Error | 0.000364 | 0.0237 | 0.1388 | 0.00692 | 0.0236 | 0.1371 |
|  | p-value | 0.00087** | 0.026* | 0.494 | 0.0001** | 0.039* | 0.499 |
|  | Adjusted R-squared | 0.109 | | | 0.0952 | | |
|  | F-statistic | 5.186 | | | 6.28 | | |
| Diabetes mellitus prevalence | Coefficient | 0.001653 | -0.1957 | 0.0839 | 0.03666 | -0.1830 | 0.0842 |
|  | Standard Error | 0.000327 | 0.0213 | 0.1247 | 0.00615 | 0.0209 | 0.1218 |
|  | p-value | 0.0** | 0.0** | 0.50 | 0.0** | 0.0** | 0.49 |
|  | Adjusted R-squared | 0.187 | | | 0.216 | | |
|  | F-statistic | 36.85 | | | 41.22 | | |
